# Dr. Paul Ehrlich

**Published:** 1915-10

**Authors:** 


**Dr. Paul Ehrlich**, noted for his work in standardizing diphtheria antitoxin, developing the side-chain theory and discovering salvarsan and neosalvarsan, died at Berlin, August 20, aged 61.

